# Beyond the Battlefield: Lymphoma Outcomes in Post-9/11 Service Members and Veterans

**DOI:** 10.3390/jcm15134973

**Published:** 2026-06-26

**Authors:** Steven J. Gibson, Matthew J. Rendo, Helen Ma, Carson Williams, Robert M. Sgrignoli, Juliana Pitzer, Joshua L. Fenderson, Wendy Cozen, James K. Aden, Sarah Darmon, Michael J. Morris, Christin B. DeStefano

**Affiliations:** 1 Brooke Army Medical Center, JB San Antonio, Ft Sam Houston, San Antonio, TX 75284, USA; steven.j.gibson24.mil@health.mil (S.J.G.); james.k.aden2.civ@health.mil (J.K.A.); michael.j.morris34.civ@health.mil (M.J.M.); 2 Wright Patterson Medical Center, Dayton, OH 45433, USA; matthew.j.rendo.mil@health.mil; 3 VA Long Beach Healthcare System, Long Beach, CA 90822, USA; helen.ma@va.gov; 4 Department of Medicine, University of California, Irvine, Irvine, CA 92697, USA; wcozen@hs.uci.edu; 5 Walter Reed National Military Medical Center, Bethesda, MD 20889, USA; carson.l.williams14.mil@health.mil; 6 Tripler Army Medical Center, Honolulu, HI 96859, USA; robert.m.sgrignoli.mil@health.mil; 7 Department of Surgery, Uniformed Services University of the Health Sciences, Bethesda, MD 20814, USA; juliana.pitzer@usuhs.edu (J.P.); sarah.darmon.ctr@usuhs.edu (S.D.); 8 The Henry M. Jackson Foundation for the Advancement of Military Medicine, Inc., Bethesda, MD 20817, USA

**Keywords:** non-Hodgkin lymphoma, mortality, deployment, military service

## Abstract

**Background**: Non-Hodgkin lymphoma (NHL) is the most common hematologic malignancy among U.S. military service members (SMs), yet the impact of deployment and deployment-related factors on survival remains unclear. Modern military service involves potential exposure to environmental hazards and trauma that could plausibly influence lymphoma biology, treatment tolerance, or long-term outcomes. **Methods**: Tricare beneficiaries with NHL diagnosed between 2001 and 2022 were identified from the Defense Health Agency Cancer Registry. Deployment history, demographics, lymphoma subtype, and mortality data were obtained from linked Department of War and National Death Index sources. Survival was compared between previously deployed (9/11 DEP) and non-deployed (NON-9/11 DEP) SMs using multivariable Cox models. Among deployed SMs, associations between deployment characteristics and mortality were evaluated. **Results**: Among 745 SMs, 378 had deployed, and 367 had not. There were no significant differences in all-cause, lymphoma-specific, or non-lymphoma mortality between cohorts. Deployment location, duration, number of deployments, and occupational category did not affect survival. Histologic differences between cohorts, higher diffuse large B-cell lymphoma and follicular lymphoma in deployed SMs, and higher T-cell lymphomas and marginal zone lymphoma in non-deployed SMs, were more consistent with unmeasured confounding and differential deployability than with deployment-related risk. **Conclusions**: These findings do not suggest major survival differences attributable to deployment status. Given the lack of publicly available validated exposure assessment tools for military personnel, as current military operations expand, including recent deployments to the Middle East, proactive and systematic approaches to exposure assessment are urgently needed. Strengthening military cancer registries and embedding validated exposure tools will be essential to proactively anticipate, identify, and manage exposure-related hematologic concerns in future Veterans.

## 1. Introduction

Mortality from cancer poses a significant risk to military operational readiness, with neoplasms remaining a leading cause of death among US military SMs [1]. B-cell non-Hodgkin lymphomas (NHL) collectively represent the most common hematologic malignancies diagnosed in active-duty service members (SMs) [2]. Risk factors for B-cell NHL include older age, male sex, immune dysregulation, chronic viral infections, and certain autoimmune conditions [3]. In comparative analyses of military and general U.S. populations, the incidence of B-cell NHL was lower among active-duty personnel, consistent with the healthier baseline status and comprehensive healthcare access characteristic of military populations [3]. Additionally, it has been shown that SMs with NHL have superior survival when compared to matched US civilians, without disparities by race or rank [4]. However, it remains unknown whether prior deployment impacts mortality from NHL.

Since 2001, nearly 3.5 million active-duty SMs have been deployed to Afghanistan or Iraq in support of Operation Enduring Freedom (OEF) [5] and Operation Iraqi Freedom (OIF) [5], and recently even more have deployed to Iran in support of Operation Epic Fury. SMs who deployed in support of OEF/OIF served in austere environments with risk of exposure to potentially carcinogenic toxins, such as in burn pits, particulate matter, jet fuel, and carcinogens released during blast injuries, among other exposures. Despite the NHL being presumptive for toxin exposure by the Veterans Health Administration [6], it has yet to be shown that deployments to locations with presumptive exposures truly alter the risk, biology, or outcomes of lymphoid neoplasms in SMs [7].

There are biological and healthcare-related mechanisms through which overseas deployment could plausibly affect survival outcomes of SMs who were later diagnosed with NHL. Severe trauma, such as that resulting in limb amputation or traumatic brain injury, has been linked to innumerable comorbidities affecting multiple organ systems, as well as heightened risk of cancer mortality [8,9,10], and could pose challenges to accurately dosing chemotherapy based on body surface area or creatinine clearance [8]. Late infections, including multi-drug resistant organisms, encountered during a deployment, could reactivate during treatment and heighten the risk of treatment complications and delays [9,10]. Additionally, survivors of war face increased mental health sequelae, such as post-traumatic stress disorder, that can lead to a heightened risk of suicide, as well as chemotherapy-induced neurologic decompensation [11]. Collectively, these physical and mental ramifications of war may pose challenges to treating hematologists when it comes to delivering curative intent therapy and may compromise eligibility for clinical trial enrollment. Among those who do receive standard care therapies, lymphoma survivors are uniquely at increased risk of death related to a dysfunctional immune system or prior therapies, such as infections or second primary malignancies.

How modern-day military service and deployment-specific factors affect NHL outcomes is unknown. Examining the impact of these characteristics is timely, as more attention and focus are placed on health promotion programs and interventions targeting SMs and Veterans, who may be at higher risk of worse outcomes.

## 2. Methods

### 2.1. Study Population and Inclusion/Exclusion Criteria

Tricare beneficiaries with NHL were identified from the Defense Health Agency Cancer Registry. All lymphoma cases were identified based on the International Classification of Diseases for Oncology (ICD-O) Histology, 3rd edition, inclusive of the lymphoid malignancies NHL and chronic lymphocytic leukemia/small lymphocytic lymphoma (CLL/SLL). Inclusion criteria were Tricare beneficiaries who had served in the military and were diagnosed with NHL or CLL/SLL between 2001 and 2022. Military service members and Veterans who served prior to the 1990–1991 Gulf War were excluded, as were dependents and other beneficiaries. Among service members who deployed, those diagnosed with lymphoid neoplasms prior to deployment were excluded.

### 2.2. Data Collection and Variables Assessed

Dates of death and causes of death were obtained from the Centers for Disease Control and Prevention’s National Death Index. The Defense Manpower Data Center was queried to obtain deployment history, including dates of deployment, deployment location, and military occupational specialty codes during deployment. Variables assessed included demographics, lymphoma subtype, tumor stage, deployment status, and deployment characteristics. Deployment variables were used as proxy indicators of potential environmental or operational exposures, as no validated individual-level exposure measurements are available in the linked datasets. Because deployment does not capture specific toxicants, intensity, or duration of exposure, these proxy measures are subject to exposure misclassification.

### 2.3. Statistical Analysis

The primary objectives were: (1) to compare the survival of military personnel with lymphoid malignancies who previously deployed versus those who never deployed, and (2) to assess the impact of deployment variables on survival among those who previously deployed. Categorical data were summarized using percentages and analyzed using the chi-squared statistic. Means and standard deviations were used for continuous variables and analyzed using Student’s *t*-test. Cox proportional hazards regression models were used to estimate hazard ratios (HRs) and 95% confidence intervals (95% CI). Models were adjusted for age at diagnosis, sex, race, ethnicity, active-duty status, military branch, tumor stage, and lymphoma subtype. Analyses were conducted using SAS 9.4 (SAS, Inc., Cary, NC, USA).

### 2.4. Ethical Approval and Consent

The study was conducted in accordance with the Declaration of Helsinki and was exempt from full Institutional Review Board review under the 2018 Revised Common Rule exemption category 4. A waiver of HIPAA authorization was approved in accordance with 45 CFR 164.512.

## 3. Results

### 3.1. Demographics

Baseline demographics are shown in Table 1. A total of 745 military personnel were included, of whom 378 had previously deployed (9/11 DEP), and 367 had not previously deployed (NON-9/11 DEP). Deployed and non-deployed cohorts were balanced regarding sex, ethnicity, active-duty vs. Veteran status at the time of lymphoid neoplasm diagnosis, and branch of service. Compared to the NON-9/11 DEP cohort, the 9/11 DEP cohort had a slightly older age at diagnosis (mean age 41.7 +/− 10.6 years vs. 39.4 +/− 10.9 years), were more likely to have white race, and were more likely to have diffuse large B-cell lymphoma (DLBCL) and follicular lymphoma (FL) and less likely to have T-cell lymphoma and marginal zone lymphoma (MZL). Median follow-up was 5.3 years (range 0–20.5).

### 3.2. Survival Outcomes by Deployment Status

Between the 9/11 DEP and NON-9/11 DEP cohorts, there was no significant difference in all-cause mortality, lymphoma-specific mortality, or non-lymphoma mortality, as shown in Table 2. Death from any cause occurred in 9% (34/378) of the 9/11 DEP cohort after a median time of 2.8 years (IQR 1.0–5.4), and in 8.2% (30/367) of the NON-9/11 DEP cohort after a median time of 2.3 years (IQR 1.0–4.8), respectively (adjusted HR 1.13, 95% CI 0.61–2.10). Death from NHL or CLL/SLL occurred in 5.6% (21/378) of the 9/11 DEP cohort after a median time of 1.9 years (IQR 1.0–5.6) and in 4.9% (18/367) of the NON-9/11 DEP cohort after a median time of 2 years (IQR 0.8–2.8), respectively (adjusted HR 1.36, 95% CI 0.62–3.00). There were no clinically significant differences in death from secondary cancers, cardiovascular disease, external causes, or other causes between cohorts (Table 2).

### 3.3. Survival Outcomes by Deployment Characteristics

Among the 9/11 DEP cohort, none of the deployment characteristics assessed impacted all-cause mortality (Table 3). In terms of deployment location, compared to deployment to Iraq, deployment to Afghanistan (adjusted HR 1.62, 95% CI 0.51–5.13), to Iraq and Afghanistan (adjusted HR 2.84, 95% CI 0.66–12.22), or to other locations (adjusted HR 0.96, 95% CI 0.29–3.19) did not increase mortality risk. Serving on 2 deployments (adjusted HR 1.89, 95% CI 0.68–5.26) or 3 or more deployments (adjusted HR 1.04, 95% CI 0.36–3.00) did not increase mortality risk over a single deployment. Compared to 1–2 locations per deployment, 3–5 locations (adjusted HR 1.01, 95% CI 0.36–2.85) and 6 or more locations (adjusted HR 1.58, 95% CI 0.53–4.73) did not affect mortality risk. Occupation category during deployment, including infantry/battle, engineering/maintenance, communication/intelligence, healthcare, multiple occupations, and other occupations, also did not affect all-cause mortality.

Among the 9/11 DEP cohort, most lymphoid neoplasms were diagnosed 10+ years after deployment (133/378, 35.2%), followed by 5–10 years after deployment (111/378, 29.4%), 1–4 years after deployment (109/378, 28.8%), and 0–1 years after deployment (25/378, 6.6%). All-cause deaths by latency after deployment are shown in Table 4. In adjusted Cox models incorporating these latency categories, there were no statistically significant differences in all-cause mortality across intervals (0–1 years: aHR 0.17, 95% CI 0.02–1.33; 1–4 years: aHR 1.32, 95% CI 0.39–4.49; 5–10 years: aHR 1.49, 95% CI 0.44–5.08; ≥10 years: reference). NHL-specific mortality results were similar, with no significant differences across latency categories.

## 4. Discussion

As warfare has evolved over the centuries, so have the immediate and long-term health ramifications of war. In modern conflicts with countries in the Middle East, SMs currently or previously faced potential exposures to hazards from burn pits, particulate matter, and jet fuel, among other exposures [6]. While the relationship between these exposures and cancer risk remains difficult to characterize, mortality is a critical endpoint in understanding long-term health outcomes. Not only are publicly available validated exposure assessments lacking for individual use and absent in military cancer registries, but exposure assessment is very challenging to ascertain due to recall bias, survivorship bias, variations in exposure definition (“jet fuel” vs. specific constituents), variability in exposure intensity and duration, individual variability in metabolism of an exposure, and confounding exposures before and after military service. Therefore, we characterized deployment and deployment variables as proxies for exposures or other operational stresses (trauma, psychological stress, etc.) using the best available and accurate data source of the Defense Manpower Data Center. In this study, we evaluated all-cause, lymphoma-specific, and non-lymphoma mortality among SMs and Veterans diagnosed with NHL, comparing those who previously deployed to those who had not. After adjustment for potential confounders, prior deployment did not affect survival. Additionally, among SMs who deployed, deployment location, duration, number of deployments, and deployment occupation did not affect mortality.

The Promise to Address Comprehensive Toxics Act qualifies NHL as a presumptive condition for toxin exposure [12], but is not limited to Veterans with neither specified job codes nor deployment-specific tasks. Additionally, there is currently no objective, standardized, and validated method to capture and quantify clinically significant exposures during military service, representing a critical unmet need for both active-duty service members and Veterans. Although it is possible that certain deployment durations, locations, and occupations put SMs at varying risks of exposures or lifestyle modifications that might contribute to lymphoma risk, our analysis did not find any association between deployment variables and mortality. The absence of increased lymphoma lethality in deployed SMs, despite adjusting for critical confounders, indicates that survival outcomes do not differ by deployment status; however, biologic differences that do not affect mortality cannot be excluded.

Differences in NHL histologies between the 9/11 DEP and NON-9/11 DEP cohorts emerged as a notable descriptive finding, with deployed SMs demonstrating higher proportions of DLBCL and FL, and non-deployed SMs showing higher proportions of T-cell lymphomas and MZL. These patterns warrant cautious interpretation, as histologic comparisons were not a primary endpoint, and our dataset lacks validated exposure information. Importantly, these differences occur within the context of the well-described healthy deployer effect and broader deployability bias: individuals selected for deployment are generally healthier, fitter, and more operationally capable, whereas non-deployed SMs may include those with chronic medical conditions, autoimmune disease, inflammatory states, or other factors that reduce deployability. The two-year age difference between cohorts is clinically negligible and unlikely to explain these patterns. The higher proportion of FL in the deployed cohort may reflect, but does not confirm, increased CT imaging during and after deployment, and should be interpreted as a hypothesis rather than a demonstrated mechanism [13]. In contrast, the NON-9/11 DEP cohort’s higher proportion of T-cell lymphomas may be influenced, but is not proven to result from, its larger proportion of Black SMs, a population disproportionately affected by peripheral T-cell lymphoma [14] and cutaneous T-cell lymphoma [15]. The greater number of MZL cases in the non-deployed cohort could also plausibly relate to, but is not confirmed to reflect, chronic antigenic stimulation through underlying chronic disease [16] that limited deployability. For both T-cell lymphomas and MZL, reverse causality is plausible: these histologies, or the chronic conditions associated with them, may reduce the likelihood of deployment rather than deployment influencing lymphoma subtype. Therefore, it is unlikely that deployment contributed to differences in histology; rather, these patterns are more plausibly explained by unmeasured confounding when comparing deployable and non-deployable SMs. These proposed explanations should be viewed as hypotheses that require validation in future studies.

Given the substantial differences in lymphoma subtype distribution between deployed and non-deployed cohorts, we conducted post hoc, subtype-stratified survival analyses to assess whether these imbalances influenced our findings. Separate Cox models within DLBCL, FL, and T-cell lymphoma demonstrated no statistically significant differences in all-cause mortality (DLBCL: aHR 0.39, 95% CI 0.14–1.05; FL: aHR 0.83, 95% CI 0.12–5.87; T-cell lymphoma: aHR 1.88, 95% CI 0.55–6.41) or NHL-specific mortality (DLBCL: aHR 0.39, 95% CI 0.12–1.35; FL: aHR 0.49, 95% CI 0.01–25.01; T-cell lymphoma: aHR 1.88, 95% CI 0.43–8.25). Although confidence intervals were wide due to limited events, these analyses indicate that the overall null association between deployment and mortality is not driven by subtype imbalance.

Stage at diagnosis also differed substantially between cohorts, with deployed service members more often presenting with stage III–IV disease and non-deployed service members more frequently diagnosed at stage I. To further evaluate this imbalance, we conducted a post hoc ordinal logistic regression restricted to the three most common subtypes (DLBCL, FL, and T-cell lymphomas), as case counts for other histologies were too small for meaningful comparison. In our primary analysis, stage was modeled as a categorical variable (stages I, II, III, IV, or unknown). In our post hoc analysis, patients with unknown stage were excluded to allow a direct assessment of stage distribution. Deployed service members were significantly more likely to present with later-stage disease for FL (aOR 4.90; 95% CI 2.19–11.00) and T-cell lymphomas (aOR 11.19; 95% CI 3.62–34.56), whereas stage differences for DLBCL were not statistically significant (aOR 1.49; 95% CI 0.86–2.59). We also examined stage-specific survival. Stage 2 could not be modeled due to small numbers, and hazard ratios for stages 1, 3, and 4 were imprecise and non-significant. Potential explanations for the observed stage imbalance include delayed healthcare access, survivorship bias, healthcare utilization differences, differential surveillance, or underlying biologic differences. Taken together, these findings indicate that stage imbalance varies by subtype and that neither stage distribution nor stage-specific survival suggests a deployment-related biological effect.

There are noteworthy limitations to this study. Although this study did not identify an association between deployment and mortality, its interpretability is limited by the small cohort drawn solely from the DHA Cancer Registry and by the absence of validated exposure data as well as treatment information. Mortality is directly influenced by treatments received, including chemotherapy regimens, stem cell transplantation, novel immunotherapeutics, and treatment intensity. These treatment variables are not available in the cancer registry and, therefore, were not assessed. Therefore, it is unknown whether military personnel who have previously deployed are treated differently from those with no prior deployment. In addition, the relatively small number of mortality events limits the ability to detect modest differences in survival. Future outcomes-focused studies of military personnel with hematologic malignancies should integrate cancer registry data across the DHA, VHA, and state registries to more completely capture cases and survival along the active-duty to Veteran continuum. Validated, clinically meaningful exposure assessments are urgently needed in military healthcare and should be embedded directly within military cancer registries and other electronic-health record data to support accurate risk estimation and strengthen scientific rigor.

In summary, our findings do not suggest major survival differences attributable to deployment status. While this finding is historically consistent with the healthy deployer effect [17], it also underscores how little is understood about the heterogeneous factors that shape survival after military service. Sustained physical and psychological trauma, quality of life burdens, infection risk, treatment-related toxicity, and unmeasured exposures may all influence long-term outcomes in ways not captured by current data systems. With ongoing operations and escalating conflict in the Middle East, the anticipation and management of service-related hematologic concerns in future Veterans will require more comprehensive surveillance, integrated data systems, and validated exposure assessments capable of capturing the true complexity of modern military service.

## Figures and Tables

**Table 1:** Baseline demographics.

	NON-9/11 DEP (N = 367)	9/11 DEP (N = 378)	*p*-Value
**Age at diagnosis (years)**	39.4 ± 10.9	41.7 ± 10.6	0.0030
**Sex, N (%)**			0.5352
Men	327 (89.1)	342 (90.5)	
Women	40 (10.9)	36 (9.5)	
**Race, N (%)**			0.0013
White	237 (64.6)	290 (76.7)	
Black	72 (19.6)	47 (12.4)	
Other	58 (15.8)	41 (10.8)	
**Ethnicity, N (%)**			0.1861
Non-Hispanic	282 (78.1)	314 (83.3)	
Hispanic	31 (8.6)	27 (7.2)	
Unknown	48 (13.3)	36 (9.5)	
**Active Duty, N (%)**			0.5980
Yes	278 (75.7)	280 (74.1)	
No (Veteran)	89 (24.3)	98 (25.9)	
**Branch of Service, N (%)**			0.1681
Army	117 (31.9)	133 (35.2)	
Air Force	101 (27.5)	76 (20.1)	
Navy	76 (20.7)	78 (20.6)	
Marines	26 (7.1)	34 (9)	
Other/Unknown	47 (12.8)	57 (15.1)	
**NHL Subtype, N (%)**			<0.0001
DLBCL	118 (32.2)	147 (38.9)	
FL	44 (12)	154 (40.7)	
T-cell	117 (31.9)	24 (6.4)	
MZL	56 (15.3)	13 (3.4)	
BL	15 (4.1)	16 (4.2)	
MCL	8 (2.2)	12 (3.2)	
PMBL	9 (2.5)	2 (0.5)	
CLL/SLL	0 (0)	5 (1.3)	
THRLBL	0 (0)	5 (1.3)	
**Stage at Diagnosis, N (%)**			<0.0001
Stage I	130 (35.4)	55 (14.6)	
Stage II	49 (13.4)	58 (15.3)	
Stage III	11 (3)	82 (21.7)	
Stage IV	49 (13.4)	88 (23.3)	
Unknown	128 (34.9)	95 (25.1)	

Key: DLBCL = diffuse large B-cell lymphoma; FL = follicular lymphoma; MZL = marginal zone lymphoma; MCL = mantle cell lymphoma; BL = Burkitt lymphoma; CLL/SLL = chronic lymphocytic leukemia/small lymphocytic lymphoma, PMBL = primary mediastinal B-cell lymphoma; THRLBL = T/histiocyte-rich large B-cell lymphoma.

**Table 2:** Mortality among SMs by Deployment Cohort.

	NON-9/11 DEP (N = 367)		9/11 DEP (N = 378)			
	N (%)	Median Time-to-Death (Years, IQR)	N (%)	Median Time-to-Death (Years, IQR)	HR (95% CI)	aHR (95% CI) ^a^
**All-cause mortality**	30 (8.2)	2.3 (1.0, 4.8)	34 (9.0)	2.8 (1.0, 5.4)	1.16 (0.71, 1.90)	1.13 (0.61, 2.10)
**NHL-specific mortality**	18 (4.9)	2.0 (0.8, 2.8)	21 (5.6)	1.9 (1.0, 5.6)	1.19 (0.63, 2.23)	1.36 (0.62, 3.00)
**Other Mortality**	12 (3.3)	2.6 (1.3, 5.6)	13 (3.4)	2.9 (1.9, 4.6)	1.13 (0.51, 2.47)	-
Secondary cancer	3 (0.8)	2.7 (1.2, 4.7)	2 (0.5)	2.9 (2.9, 2.9)	0.69 (0.12, 4.14)	-
CV Disease	1 (0.3)	2.5 (2.5, 2.5)	3 (0.8)	4.5 (2.8, 5.4)	3.16 (0.33, 30.37)	-
External Causes	3 (0.8)	1.4 (0.5, 7.8)	2 (0.5)	9.6 (9.0, 10.1)	0.70 (0.12, 4.21)	-
Other	5 (1.4)	4.8 (2.6, 6.4)	6 (1.6)	1.4 (0.4, 2.8)	1.23 (0.38, 4.04)	-

^a^ Model adjusted for age at diagnosis, sex, race, ethnicity, active-duty status, military branch, tumor stage, and lymphoma subtype.

**Table 3:** Impact of deployment characteristics and occupation on survival in deployed SMs.

9/11 DEP (N = 378)			
	N (%)	All-Cause Mortality, N (%)	aHR (95% CI) ^a^
**OIF/OEF location**			
Iraq	80 (21.2)	8 (10.0)	1.00 (ref)
Afghanistan	65 (17.2)	9 (13.8)	1.62 (0.51, 5.13)
Iraq and Afghanistan	23 (6.1)	4 (17.4)	2.84 (0.66, 12.22)
Other	210 (55.6)	13 (6.2)	0.96 (0.29, 3.19)
**Number of deployments**			
1	125 (33.1)	8 (6.4)	1.00 (ref)
2	125 (33.1)	17 (13.6)	1.89 (0.68, 5.26)
3+	128 (33.9)	9 (7.0)	1.04 (0.36, 3.00)
**Deployment length**			
<6 months	63 (16.7)	8 (12.7)	1.00 (ref)
6–<12 months	68 (18.0)	3 (4.4)	0.53 (0.12, 2.24)
12–<24 months	65 (17.2)	4 (6.2)	0.64 (0.15, 2.70)
24+ months	182 (48.2)	8 (12.7)	0.83 (0.26, 2.59)
**Locations per deployment**			
1–2	138 (36.5)	10 (7.2)	1.00 (ref)
3–5	104 (27.5)	10 (9.6)	1.01 (0.36, 2.85)
6+	136 (36.0)	14 (10.3)	1.58 (0.53, 4.73)
**Occupation**			
Engineering/Maintenance	26 (6.9)	2 (7.7)	0.54 (0.11, 2.62)
Communication/Intelligence	28 (7.4)	5 (17.9)	2.94 (0.74, 11.65)
Healthcare	15 (4.0)	1 (6.7)	0.31 (0.03, 2.87)
Other	42 (11.1)	5 (11.9)	0.42 (0.11, 1.62)
Multiple Occupations	267 (70.6)	21 (7.9)	1.00 (ref)

^a^ Model adjusted for age at diagnosis, sex, race, ethnicity, active-duty status, military branch, tumor stage, and lymphoma subtype.

**Table 4:** Latency from deployment to NHL.

	Deployed (N = 378)		
	Total N (%)	All-Cause Death N (%)	aHR (95% CI) ^a^
Cancer Diagnosis			
0–1 year after deployment	25 (6.6)	2 (8.0)	0.17 (0.02, 1.33)
1–4 years after deployment	109 (28.8)	12 (11.0)	1.32 (0.39, 4.49)
5–10 years after deployment	111 (29.4)	11 (9.9)	1.49 (0.44, 5.08)
10+ years after deployment	133 (35.2)	9 (6.8)	1.00 (ref)

^a^ Model adjusted for age at diagnosis, sex, race, ethnicity, active-duty status, military branch, tumor stage, and lymphoma subtype.

## Data Availability

The original data presented in this study are available upon request from the Joint Pathology Center, Defense Manpower Data Center, and Defense Suicide Prevention Office.
